# Solvent Vibrations as a Proxy of the Telomere G-Quadruplex Rearrangements across Thermal Unfolding

**DOI:** 10.3390/ijms23095123

**Published:** 2022-05-04

**Authors:** Valeria Libera, Federico Bianchi, Barbara Rossi, Francesco D’Amico, Claudio Masciovecchio, Caterina Petrillo, Francesco Sacchetti, Alessandro Paciaroni, Lucia Comez

**Affiliations:** 1 Dipartimento di Fisica e Geologia, Università degli Studi di Perugia, 06123 Perugia, Italy; valeria.libera@studenti.unipg.it (V.L.); federico.bianchi86@outlook.it (F.B.); caterina.petrillo@unipg.it (C.P.); francesco.sacchetti@unipg.it (F.S.); 2 IOM-CNR c/o Department of Physics and Geology, Università degli Studi di Perugia, 06123 Perugia, Italy; 3 Elettra Sincrotrone Trieste, S.S. 14 Km 163.5, 34012 Trieste, Italy; barbara.rossi@elettra.eu (B.R.); francesco.damico@elettra.eu (F.D.); claudio.masciovecchio@elettra.eu (C.M.)

**Keywords:** G-quadruplex, hydrogen bonding, resonant Raman spectroscopy, circular dichroism, hydration water

## Abstract

G-quadruplexes (G4s) are noncanonical forms of DNA involved in many key genome functions. Here, we exploited UV Resonance Raman scattering to simultaneously explore the vibrational behavior of a human telomeric G4 (Tel22) and its aqueous solvent as the biomolecule underwent thermal melting. We found that the OH stretching band, related to the local hydrogen-bonded network of a water molecule, was in strict relation with the vibrational features of the G4 structure as a function of temperature. In particular, the modifications to the tetrahedral ordering of the water network were strongly coupled to the DNA rearrangements, showing changes in temperature that mirrored the multi-step melting process of Tel22. The comparison between circular dichroism and Raman results supported this view. The present findings provide novel insights into the impact of the molecular environment on G4 conformation. Improving current knowledge on the solvent structural properties will also contribute to a better understanding of the role played by water arrangement in the complexation of G4s with ligands.

## 1. Introduction

In molecular biology, guanine-rich sequences of nucleic acids can fold into four-stranded, non-canonical secondary structures called G-quadruplexes (G4s). G4s were initially considered as a rather rare structural novelty, but recent discoveries suggest their involvement in key genome functions, such as transcription, replication, epigenetic regulation, and genome stability, with the following numerous connections to cancer biology [1,2,3,4,5]. As a whole, these discoveries stimulated a huge body of research to probe G4 functional mechanisms and the consequent opportunities for therapeutic intervention.

The G-tetrad, a cyclic Hoogsteen hydrogen-bonding arrangement of four guanines with each other, is the building block of G4s. The quadruplex stem is composed of stacked G-tetrads with phosphodiester backbones delimiting cavities called grooves. The formation of G4s is driven by monovalent cations such as Na+ and K+; hence physiological buffers favor their assembly [6,7]. G4s can be unimolecular or intermolecular and can adopt a wide diversity of topologies arising from different combinations of strand direction, as well as sequence length and loop (the chains linking the strands) composition. A systematic classification of G4 topologies can be made by exploiting the glycosidic bond angle of the intervening bases, which can assume either an anti or a syn arrangement [8]. Different combinations of these units, i.e., anti–anti, syn–anti, anti–syn, or syn–syn, give rise to parallel and anti-parallel strand orientations [9,10,11]. Hybrid conformations composed of mixtures of parallel and anti-parallel strands are also allowed [12,13], and several classifications have been reported in the literature [13,14].

The G4 assembling process is mainly governed by non-covalent interactions of individual nucleosides and their aqueous environment. A key contribution to the structural stability is given by the hydrophobic stacking interactions between guanine bases [15]. The stacking directionality and orientation are affected by the backbone steric constraints and electrostatic interactions involving oligonucleotides and their environment. The latter is a crucial point to be explored, as it has been demonstrated that waters that are in close contact with biomolecules (i.e., hydration water layers) play a key role in the folding, stability, and function. On the other hand, due to mutual solute–solvent interactions, both the structural organization and mobility of solvent in close proximity to biological macromolecules differ from bulk solvent [16,17,18,19,20,21].

In the case of duplex DNA, one of the most well-known hydration-dependent effects is the structural transition between the A- and B-form [22]. It is also acknowledged that changes in the hydration shell may alter the DNA shape in a sequence-dependent way [23,24,25,26,27,28,29]. Unlike duplex DNA, which is characterized by two wide and narrow grooves, G4s have four grooves, each held together by phosphodiester chains. Depending on the G4 topology, these grooves have varying widths. In parallel quadruplexes, they are all of medium extent, while hybrid and anti-parallel structures may have three types of grooves: wide, medium, and narrow. Host water molecules can fill both the narrow and medium grooves, but it was recently shown that only the narrow one can accommodate extended filiform networks of water molecules [30]. These water channels, by analogy with the water arrangement in the minor groove regions of duplex DNA, were referred to as spines. It follows that anti-parallel and hybrid structures can host more stable water spine structures than parallel ones. Moreover, there is evidence that, under crowded conditions, G4s are more mobile than DNA duplexes and more prone to changing their conformational state [31,32,33]. Such a structural polymorphism of G4s was found to be strictly regulated by their state of hydration [34,35,36,37,38,39].

Less studied is the relation between the polymorphic nature of quadruplexes and their solvation attitude in diluted aqueous solutions. In these conditions, the different folds can be separated by relatively small energy barriers, and the switching between distinct conformers may occur fairly easily under the action of external parameters, such as temperature, pressure, and ionic strength [6]. Additionally, the unfolding pathway generally occurs through intermediate stages, in response to even small environmental variations [40,41]. In this context, the connection between the rearrangement of the H-bond network in G4 solutions and the G4 conformational switching upon unfolding deserves to be studied. An unrivaled technique suitable for this purpose is Ultra Violet Resonance Raman (UVRR) spectroscopy [42], in which the UV excitation wavelength is in resonance with DNA electronic transition [43,44,45,46]. This provides both chromophore selectivity and special sensitivity for label-free recognition of a chromophore moiety of the molecule even at biologically relevant micromolar concentrations. The Raman signal is, in fact, selectively enhanced by a few to several orders of magnitude. Such an enhancement allows for the investigation at the same time of the vibrational markers of the G4 and of the solvent, making it possible to combine information from Raman experiments with those from techniques especially suited for highly diluted systems. One of these techniques is circular dichroism (CD), which is an excellent tool for rapid determination of the secondary structure and folding properties of biomolecules.

Here, we show how UVRR and CD spectroscopies can be effectively combined to provide insights into structural and molecular aspects of the interaction between G4s and their environment upon thermal unfolding. Among the G4 families, we focused on the archetypal human telomeric sequence d[AG_3_(TTAG_3_)_3_], consisting of 22 nucleobases (Tel22), that, in a K+ environment and at ambient temperature, is formed by a mixture of hybrid and anti-parallel topologies [47,48]. Several techniques have already been employed to study the structure and thermodynamics of Tel22 solutions [49,50,51,52,53]. Singular value decomposition (SVD) analysis [53,54,55,56] applied to experimental data revealed that Tel22 undergoes melting through a multi-state process, i.e., by populating different intermediate states in a sequential manner. In this study, we used a multivariate method to analyze both solute and solvent UVRR fingerprint regions, and then related vibrational features with secondary structure characteristics elucidated by CD.

## 2. Results

### 2.1. A Broad Band UVRR Data Treatment

**Figure 1:**
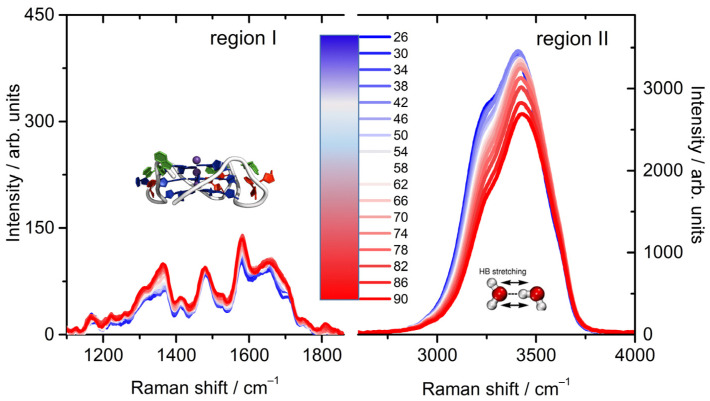
UVRR spectra of the Tel22 K+ water solution at 45 μM as a function of temperature. Colors from blue to red indicate the Raman profiles recorded from 26 °C to 90 °C with steps of 4 °C.

The potential of UVRR spectroscopy is here exploited by using the excitation wavelength λ_exc_ = 220 nm so as to separately enhance the chromophore-specific vibrations from a dG, dT, and dA basis [57,58], simultaneously acquiring the intense broad OH stretching region in the same spectrum. The whole collection of UVRR spectra of the Tel22 water solution, over the huge wavenumber range (1000–4000 cm^−1^), as a function of temperature is represented in Figure 1, where two main regions are separately enhanced: the first (I) over the 1300–1800 cm^−1^ range, mainly due to the Tel22 vibrations, and the second (II) over the 3000–3900 cm^−1^ range, related to the intramolecular structure of the H-bond network. The strength and novelty of the present work, therefore, resides in its ability to (1) jointly follow the trend of the vibrational features of both the solute and the solvent in a wide spectral profile with the high selectivity of the UVRR probe, and (2) to apply a bidimensional analysis over the Raman fingerprint regions to investigate whether or not those vibrations are correlated as the temperature is increased.

The very first step of the analysis was to apply the SVD method to the UVRR spectra of the Tel22 aqueous solution shown in Figure 1. SVD is a model-free analytical tool widely used to analyze data where the experimental response is a function of two quantities, temperature and wavenumber in our case (further details are given in the Supplementary Materials). Application of SVD to the present three-dimensional UVRR data provides two sets of eigenvectors, one depending on temperature and the other on wavenumber (Figure S1 in the Supplementary Materials). By using an acceptance/rejection criterion on eigenvectors and eigenvalues, it is then possible to determine the number of significant spectral species, Ns, able to reproduce the significant changes in the experimental profiles with the increasing temperature.

To determine such a number, the autocorrelation of the eigenvectors, as well as the magnitude and the percentage variance of the eigenvalues must be screened by referring to a cut-off level defined on a statistical basis [55]. The SVD protocol applied to the UVRR data of Figure 1 in the region I (selected range: 1390–1800 cm^−1^), over the measured temperature range, showed evidence for three spectroscopically distinct species (Ns = 3, Table S1 in the Supplementary Materials). The significant vectors were then globally fit by iterative nonlinear least-squares to the analytical expressions for thermal unfolding corresponding to the two-step sequential pathway F⇌ I⇌ U (Figure S2). The global equation (Equation (3), in Materials and Methods) is based on a van’t Hoff analysis of multiple thermal transitions [56], in analogy with change in state functions of proteins. These SVD results, complementing those obtained using a 250 nm excitation wavelength [51] where vibrational bands were associated with different UV absorbing chromophores [58], support the evidence that the molecular vibrations related to stretches of ring bonds of a nucleotide basis experience a multistep thermal path towards unfolding.

**Figure 2:**
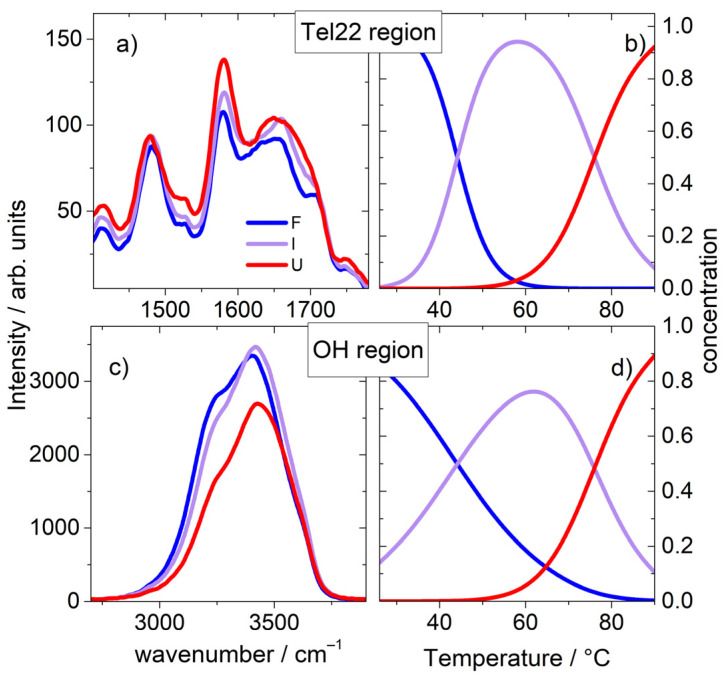
Results of the SVD analysis on UVRR data of Figure 1. Left panels: spectra of significant species for Tel22 (**a**) and OH stretching region (**c**). Right panels: relative concentration of significant species as function of temperature for Tel22 (**b**) and OH stretching region (**d**). Blue, lilac and red colors correspond to folded, intermediate and unfolded states, respectively.

After this first step, we progressed with the SVD analysis over the OH stretching region (zone II, Figure 1), mainly attributed to water vibrations. Remarkably, we found that the F-I-U pattern is still valid, suggesting a connection between solvent and solute vibrational modes. We thus proceeded with a global fit of the two sets of data (I and II) using Equation (3) and by sharing the T_m1_ and the T_m2_ parameters. Through this, we were able to identify the transitional states in correspondence with the pre-melting and melting temperatures equal to T_m1_ (44 °C) and T_m2_ (76 °C), respectively. The whole set of thermodynamic parameters is reported in Table S2 in the Supplementary Materials. The significant species and relative concentrations are shown in Figure 2a–d.

### 2.2. The Fingerprint Region of Tel22 Cromophores

**Figure 3:**
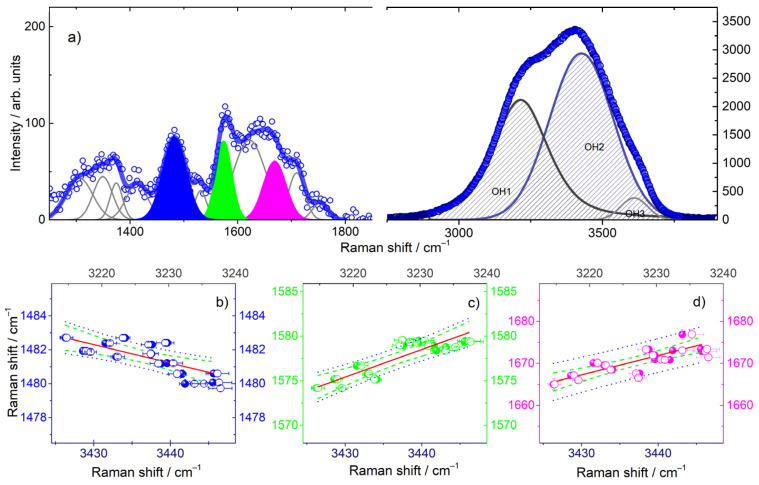
Panel (**a**) UVRR spectra of the Tel22 K+ water solution at 45 μM at ambient temperature compared to the single and cumulative fitting curves. Panels (**b**) refer to the plot of ν_1482_ vs. ν_OH1_ (left and bottom) and of ν_1482_ vs. ν_OH2_ (right and top), panels (**c**) to the plot of ν_1575_ vs. ν_OH1_ (left and bottom) and of ν_1575_ vs. ν_OH2_ (right and top), and finally panels (**d**) to the plot of ν_1670_ vs. ν_OH1_ (left and bottom) and of ν_1670_ vs. ν_OH2_ (right and top). The red lines represent a fit to a straight line. The corresponding Pearson’s *r* values were −0.75 and −0.85 for data in the panel (**b**), 0.89 and 0.87 for the data in the panel (**c**), and 0.83 and 0.84 for the data in the panel (**d**); 95% confidence (green) and prediction (blue) bands are also shown for sake of completeness. The data set indicates a good correlation between G4 and OH vibrations.

The unprecedented use of SVD analysis in such a wide spectral range points to the important finding that there is a coupling between solute and solvent vibrations upon thermal unfolding. To provide further insights on this hypothesis, we performed a quantitative analysis of the UVRR spectra, based on literature reporting the assignment of Raman bands [51,53,57,59,60,61]. The vibrational features of zone I were fitted by using a set of Gaussian functions (Figure 3a), bringing attention to the temperature evolution of the peaks centered at about 1482, 1575 and 1666 cm^−1^ (Figure 3a), attributed to out of phase stretches of the dT ring bonds coupled to the C2 carbonyls and to C5H3, to dA N6H scissoring (and, to a lesser extent, to stretching of the triene system C2 = N3-C4 = C5-N7 = C8 of dG) and to the stretching of carbonyl moieties of dG residues (C6 = O), respectively [58,62]. This analysis shows a downshift in the ν_1482_ group band vibration on increases in the temperature, compatible with a change in the C5-H bond strength, and an upshift in the ν_1575_ and ν_1680_ vibrations, which implies a strengthening variation in the N6-H and C6 = O bonds, respectively, as conformational changes proceeded. These trends (Figure S3) are also consistent with a loss of H-bonding as the temperature was increased.

Given the low concentration of Tel22 in the buffer solution (Tel22 45 μM), to reproduce the UVRR profile of the solution over zone II, we used the same methodology adopted for water in diluted solutions [63]. In these cases, the Raman profile can be represented by means of three distinct contributions, centered at about ν_OH1_ ≈ 3200 cm^−1^, ν_OH2_ ≈ 3450 cm^−1^, and ν_OH3_ ≈ 3600 cm^−1^ (Figure 3a, right side). The first component is representative of the so-called connective water, where the OH oscillators are phase-correlated with oscillators of the closest molecules. This vibration originates from ice-like tetrahedral water arrangements [64,65]. The middle feature is assigned to close water structures, where H-bonds are partially distorted and the phase correlation to vibrations of the nearest OH groups is lost [66]. The bump located at higher wavenumbers is associated with OH groups weakly stabilized by H-bond interactions. These groups can be regarded as transient species formed during the H-bond reorganization of the network [66,67]. A representative comparison between experimental and theoretical curves is provided in Figure S4 of the Supplementary Materials for both the Tel22 solution spectrum and the corresponding buffer at ambient temperature. The fitting results show an increasing trend in the ν_OH1_ and ν_OH2_ frequency bands (Figure S5) that agrees with a weakening of the hydrogen bonding as the Tel22 structure approached the unfolding.

Altogether, the results of the data analysis performed over zones I and II of the spectra allowed us to obtain information on the trend as a function of the temperature of a set of group vibrations that are relevant in the melting process. Interestingly, we identified two kinks in the temperature behavior of the frequency position, related to both specific Tel22 vibrations (ν_1482_, ν_1575_ and ν_1660_) and OH stretching of close water structures (bands 1 and 2, i.e., ν_OH1_ and ν_OH2_). These kinks were fairly in correspondence with the T_m1_ and T_m2_ determined through the SVD analysis. Therefore, we investigated whether the changes in the former (ν_1482_, ν_1575_ and ν_1660_) were followed by a change in the latter (ν_OH1_ and ν_OH2_). From Figure 3b–d, it is apparent that all the data placed in relation show a linear dependence when represented as one against the other. In particular, by performing a fit of ν_1482_ vs. ν_OH1_ (and vs. ν_OH2_), ν_1575_ vs. ν_OH1_ (and vs. ν_OH2_), and ν_1660_ vs. ν_OH1_ (and vs. ν_OH2_), we obtained Pearson’s *r* values that were all above 0.75, indicating a quite good correlation between group vibrations of the G4 bases and OH stretching vibrations. This is a sign of the intimate correlation between solute and solvent molecular vibrations along the thermal pathway.

### 2.3. The OH Stretching Band

**Figure 4:**
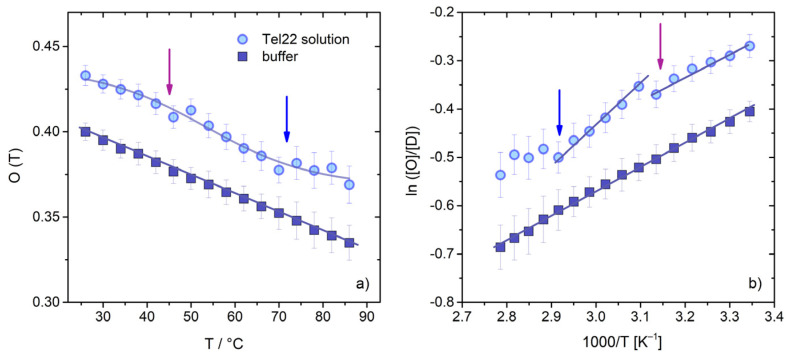
(**a**) Temperature evolution of the parameter O(T), which is the ratio between the area of the OH stretching component at a lower wavenumber, I_νOH1_(T), and the total area of the OH stretching band, I_OHtot_(T). Circles are the results for Tel22 solution, squares for the buffer. Two inflection points were identified for Tel22 data at about T’_m1_ = 44 °C and T’_m2_ = 73 °C; the continuous line is only a guide for the eye. (**b**) Van’t Hoff plot for the O↔D equilibrium for the Tel22 water solution at 45 μM at ambient temperature, and the corresponding buffer. The quantity O/D was defined as the ratio O/(1-O) as described in the text. Arrows are guides for the eyes, approximatively corresponding to T’_m1_ and T’_m2_.

A measure of the effect of G4 on the structuring of the hydrogen bonding network can be also obtained by calculating the relative ratio between the area of the OH stretching of the connective water component, I_νOH1_(T), and the total area of the OH stretching band, I_OHtot_. This quantity, which we will define from now on as O(T), provides, in diluted solutions, an estimate of the relative amount of OH groups involved in ordered tetrahedral structures, and it can be considered as a tester of the structuring/destructuring effect on water induced by the solute [63]. By calculating the parameter O(T) for both the Tel22 aqueous solution and the buffer alone (Figure 4a), we found a decreasing linear trend in agreement with the literature [63] for the latter, and a behavior that deviated from linearity for the former. The first derivative of O(T) identified two inflection points for the Tel22 solution at about T’_m1_ = 44 °C and T’_m2_ = 73 °C, which was consistent with the melting temperatures obtained through SVD (Table S3) in correspondence with the major conformational changes in the quadruplex. This result strengthens the idea of a solute–solvent mutual interaction, and also indicates that modifications in G4 topology had an influence on the tetrahedral water network around the solute as the temperature increased. The solvent alone indeed showed a linear behavior not modulated by any structural changes.

From the analysis of the temperature dependence of the OH stretching band, it is also possible to obtain the thermodynamic parameters related to the shift from an ordered (O) to a disordered (D) water structure in the presence of G4s. Specifically, the enthalpy variation ΔH_O__↔__D_ of the process can be evaluated from the temperature dependence of the O↔D equilibrium, by means of a van’t Hoff treatment in which O(T) and 1-O(T) are the fraction of OH oscillators belonging to the O and D water species, respectively [68]. An example of the van’t Hoff plot is given in Figure 4b. Linear regression fitting ln([O]/[D]) vs. inverse temperature allowed for the calculation of the buffer enthalpy, ΔH = −1.10 ± 0.02 kcal/mol, which was in reasonable agreement with the value determined for pure water by means of UVRR (λ_exc_ 266 nm [63]). Conversely, for the Tel22 water solution, we observed a not-continuous trend as a function of temperature, characterized by a change in slope corresponding to an enthalpy variation from 1.07 ± 0.02 kcal/mol to 1.55 ± 0.06 kcal/mol in the pre-melting region. The visible variation at T’_m1_ (violet arrow) further testifies that the O↔D equilibrium was extremely sensitive to the intermediate conformational changes induced by temperature on the quadruplex secondary structure before complete unfolding.

### 2.4. The Link with the Secondary Structure

**Figure 5:**
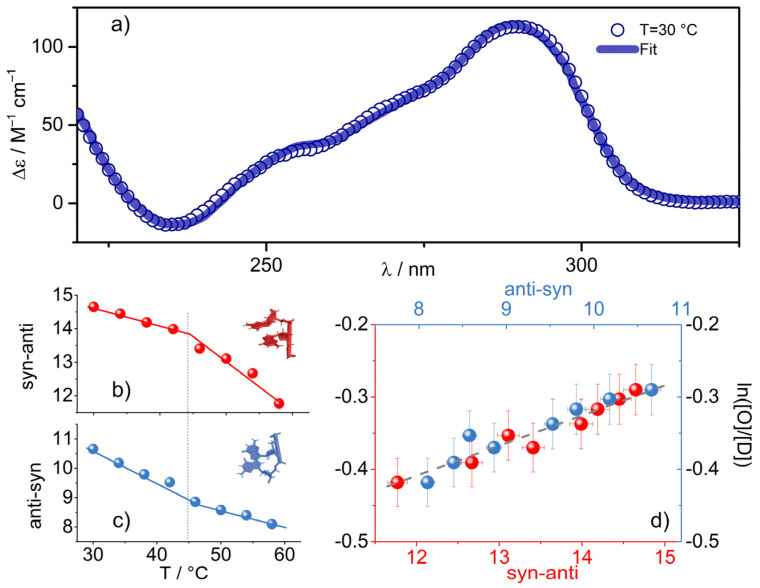
(**a**) Experimental CD profile for the Tel22 K+ water solution at 45 μM and ambient temperature, shown together with the fitted curve (solid line) obtained by using the algorithm presented in Ref. [14]. (**b**,**c**) Percentages of syn–anti and anti–syn populations as a function of temperature (dotted line approximatively corresponds to T’_m1_). (**d**) Correlation plot between syn–anti and anti–syn populations and the quantity ln([O]/[D]).

To investigate these conformational changes, we deployed the circular dichroism (CD) technique, which is a powerful method to investigate different G4 topologies. Anti–anti, syn–anti or anti–syn conformations, in diagonal or lateral loops and in other external moieties, can be derived by applying an open-source tool, based on an archive of circular dichroism spectra of 23 G-quadruplexes of known structure, defined either by X-ray crystallography or by NMR [14,69]. This algorithm was used to analyze the CD profiles of our Tel22 solutions (Figure S6 in the Supplementary Materials). The spectra were deconvoluted in terms of fold recognition in the 30–58 °C temperature range; above this temperature, the decrease in the dichroic signal associated with progressive unfolding made it impossible to apply the fitting procedure. The excellent agreement between the fit curves and experimental CD profiles can be appreciated in Figure 5a, where the spectrum at T = 30 °C is reported as an example.

We found that, despite the fact that the percentages of all populations needed to reconstruct the total CD spectrum displayed a kink in correspondence with T’_m1_ (Figure S6), as in the ln ([O]/[D]) behavior, the anti–syn and syn–anti populations were still strongly correlated with the ln ([O]/[D]) values (Figure 4b–d). We could therefore argue that the more G4 constituents are correlated with OH stretching vibration changes, the more they are involved in a mutual interaction with the solvent (Figure 5d).

## 3. Discussion

The correlation between the Tel22 secondary structure traits and the OH stretching modifications (Figure 5d) provides experimental evidence that the destructuring of the OH signal is linked not only to a temperature effect but also to a topological modulation, and in particular to a decrease in the anti-parallel population. It is worth mentioning that at room temperature, our telomeric sequence appeared to be consistent with a mixture of hybrid and antiparallel folds, while with increasing temperature, there was a progressive shift from the antiparallel [14,53] to the parallel population. This change at the secondary structure level could be responsible for a different interaction of the nucleosides with the solvent. In fact, the decrease (increase) with temperature of anti-parallel (parallel) units, involved a rearrangement of diagonal and lateral loops, and thus a different interaction of water with G4s’ loops and grooves. Although obtained in very diluted conditions and probably linked to a long-range effect, these findings are in agreement with the recent observation, obtained by examining high-resolution X-ray crystallography, that anti-parallel and hybrid quadruplex structures are able to host stable extended ordered water spines into the DNA grooves, contrary to what happens in the parallel ones [30]. To investigate this point further, it would be extremely interesting to design combined UVRR and NMR experiments, since NMR has proven particularly useful for characterizing the anti or syn conformation of dG in G4 structures in several environmental conditions [7,70,71].

Overall, we showed that in G4 aqueous solutions, as in canonical DNA helices [21,30], the solute–solvent interactions detected by Raman spectroscopy were mutual, and intriguingly, water molecular vibrations were further associated to thermally induced topological changes at the secondary structure level. Due to the enormous importance of G4s for a variety of biological functions, we believe that the unique results presented here could be a starting point for further investigations that could be carried out by varying DNA concentration, buffer ionic strength, and/or any other control parameter. Moreover, examining different G4s that are able to adopt a well-established and single topology in solution will make it possible to investigate the close relationship between the G4 conformation and the features of the extended hydrogen-bond network involving the biomolecule and its hydration water. Finally, the method described here can also be relevant for studying the importance of hydration water in the interaction of G4s with other molecules, such as ligands for therapeutical purposes, which are able to induce topological changes [72].

## 4. Materials and Methods

The oligonucleotide sequence AG_3_(TTAG_3_)_3_ was purchased from Eurogentec (Belgium) and used without further purification. The lyophilized powder was dissolved in a 50 mM phosphate buffer at pH = 7, 0.3 mM EDTA, and 150 mM KCl. This solution was heated to 95 °C for 5 min and then slowly cooled down to room temperature in ~4 h. After this procedure, the samples were left at room temperature overnight. DNA concentration was determined from UV absorption measurements at 260 nm, using a molar extinction coefficient of 228 500 M^−1^ cm^−1^ (data provided by Eurogentec). Samples for UVRR measurements were prepared at 45 μM and checked through CD measurements [73].

### 4.1. UVRR Experiments

UVRR measurements were carried out at the IUVS beamline at Elettra Sincrotrone Trieste by exploiting a properly optimized synchrotron-based experimental setup [74]. All of the samples were placed into a 10 mm path quartz cuvette for UVRR measurements. The spectra were excited at 220 nm and collected in a backscattered geometry by using a triple-stage spectrometer with a spectral resolution of about ~2.6 cm^−1^/pixel. Beam power measured on the samples was about 4 µW. For each sample, UVRR spectra were recorded in the temperature range from 26 °C to 90 °C, with steps of 4 °C.

### 4.2. CD Experiments

Circular dichroism experiments were done using Jasco J-810 spectropolarimeter on the Tel22 at 45 μM, using a 1 mm path-length quartz cuvette. Spectra were recorded in the range from 220 to 325 nm, with a scan speed of 50 nm/min, by changing the temperature from 30 to 82 °C, with steps of 2 °C via a thermal bath.

### 4.3. SVD Details

The Singular Value Decomposition (SVD) is a method to factorize a matrix, D, into the product of three matrices, U, S and V, i.e., D = U S V^T^, where V^T^ is the transpose of V.

The D matrix has as columns the UVRR experimental spectra at each temperature; the U matrix consists of the basis spectra, which combined are able to form the whole experimental dataset; S is a diagonal matrix, where the numbers on the diagonal, the singular value, represent the weights of each component. The V matrix is made up of the amplitude vectors as a function of the temperature. The method of identifying the minimum number of spectral components able to reproduce the dataset is described in [55,56]. Basically, the magnitude and the relative variance of the singular values and the autocorrelation coefficients of the vectors of the U and the V matrices must be screened according to a certain acceptance/rejection criterion. In the case of UVRR data, the SVD analysis was performed over two distinct spectral regions, namely 1390–1800 (cm^−1^) and 2700–3900 (cm^−1^), respectively called I and II. For both datasets, a cutoff of 0.65 for the autocorrelation coefficient was found to be statistically meaningful, providing the results reported in Table S1. Given this constraint, three significant V vectors were identified and associated with a folded-intermediate-unfolded (F⇆I⇄U) melting pathway [56]. Accordingly, V1–V3 were globally fitted to analytical expressions suitable for studying the thermodynamics of thermal unfolding (Figure S2, Table S2). By analogy with proteins, changes in state functions were described in terms of the van’t Hoff equations [75] briefly mentioned below.

Let us recall that for a reversible process where a biomolecule passes from a native (F) to an unfolded (U) state (e.g., F⇄U) under the action of temperature, the following equation holds to good approximation:

(1) $$\left[\varphi \right]=\frac{{\left[\varphi \right]}_{F}+K\;{\left[\varphi \right]}_{U}}{1+K}$$

where ${\left[\varphi \right]}_{F}$ and ${\left[\varphi \right]}_{U}$ are the variations in the physical observable for native (F) and unfolded (U) states, respectively, and [φ] is that which is detected in the transition region. The unfolding equilibrium constant K changes with temperature according to the van’t Hoff equation:

(2) $$\left[K\left(T\right)\right]=\exp\left[-\frac{\Delta H}{R}\left(\frac{1}{T}-\frac{1}{T_{m}}\right)\right]$$

where ∆H is the van’t Hoff unfolding enthalpy and T_m_ the denaturation temperature. As G-quadruplexes are generally characterized by multistep thermal paths, Equation (1) needs to be adapted on a case-by-case basis: in Ref. [56], several mechanisms were proposed that have to be tested on experimental datasets. For the F⇆I⇄U mechanism, which was proved to be valid for our data, it is possible to write:

(3) $$s\left(T\right)=\frac{S_{U}{\;e}^{-\frac{\mathrm{dH}1}{R}\left(\frac{1}{T_{m1}}-\frac{1}{T}\right)-\frac{\mathrm{dH}2}{R}\left(\frac{1}{T_{m2}}-\frac{1}{T}\right)}+S_{I\;}e^{\frac{{\mathrm{dH}}_{1}}{R}\left(\frac{1}{T_{m2}}-\frac{1}{T}\right)}+S_{F}}{e^{-\frac{\mathrm{dH}1}{R}\left(\frac{1}{T_{m1}}-\frac{1}{T}\right)-\frac{\mathrm{dH}2}{R}\left(\frac{1}{T_{m2}}-\frac{1}{T}\right)}+e^{\frac{{\mathrm{dH}}_{1}}{R}\left(\frac{1}{T_{m2}}-\frac{1}{T}\right)}+1}$$

where dH_i_ = dH_(folding)_ for step I; T_mi_ = mid-point temperature for step I (with I = 1 corresponding to the F↦I step and I = 2 to the I↦U step); S_F_ = optical signal for folded conformers; S_I_ = optical signal for the intermediate species; S_U_ = optical signal of the unfolded ensemble; R = 1.987 cal K^−1^ mol^−1^.

## Data Availability

Not applicable.

## Supplementary Materials

The following supporting information can be downloaded at: https://www.mdpi.com/article/10.3390/ijms23095123/s1.
