# Supplementary material for: Solvent Vibrations as a Proxy of the Telomere G-Quadruplex Rearrangements across Thermal Unfolding
Source: Int J Mol Sci. 2022 May 4;23(9):5123. doi: 10.3390/ijms23095123 (PMC9100830; doi:10.3390/ijms23095123)
Supplement: Supplementary file 1 [file ijms-23-05123-s001.zip › ijms-1685208-supplementary.pdf]

International Journal of *Molecular Sciences*

MDPI

Electronic Supplementary Information

Solvent vibrations as a proxy of the telomere G-quadruplex rearrangements across thermal unfolding

Valeria Libera[1,2], Federico Bianchi[1], Barbara Rossi[3], Francesco D'Amico[3], Claudio Masciovecchio[3], Caterina Petrillo[1], Francesco Sacchetti[1], Alessandro Paciaroni[1,*] and Lucia Comez[2,*]

[1]*Dipartimento di Fisica e Geologia, Università degli Studi di Perugia, 06123 Perugia, Italy*
[2]*CNR-IOM Istituto Officina dei Materiali c/o Dipartimento di Fisica e Geologia, Università degli Studi di Perugia, 06123 Perugia, Italy*
[3]*Elettra Sincrotrone Trieste, S.S. 14 Km 163.5, 34012 Trieste, Italy*

**corresponds to: alessandro.paciaroni@unipg.it, comez@iom.cnr.it*

Contents:

Table S1 contains the results from SVD Analysis of Melting UVRR Data for Tel22 aqueous solution.
Table S2 contains the thermodynamics parameters for the thermal melting of Tel22 aqueous solution obtained from a global fit on SVD.
Figure S1 gives an example of the multidimensional melting data and the structure of data sets used for SVD analysis.
Figure S2 shows SVD analysis of UVRR data set for Tel22 K^+ aqueous solution, over the region I and II of Figure 1 of the manuscript.
Figure S3 gives UVRR results for Tel22 solution over the region I of Figure 1 of the manuscript.
Figure S4 gives an example of the fit over the region II of the Figure 1 of the manuscript.
Figure S5 gives the result for the Raman shift of the OH stretching group bands.
Figure S6 gives CD results in terms of secondary structure principal basis.

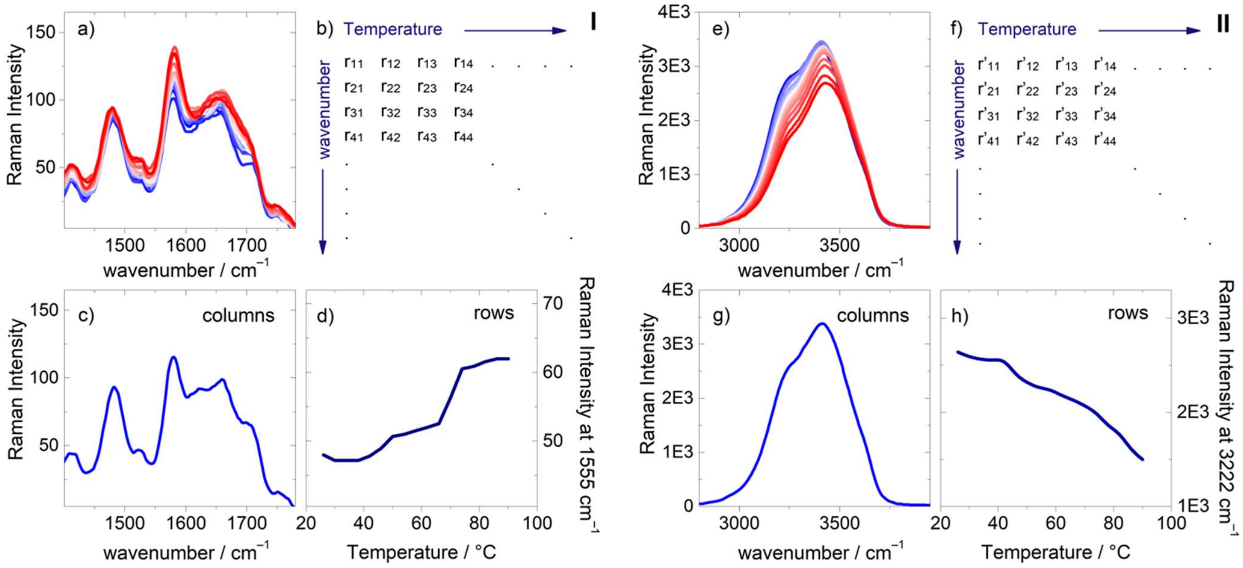

Figura S1 Multidimensional melting data and the structure of data sets used for analysis. Examples of UVRR spectra, over the region I and II of Figure 1 of the manuscript, as a function of temperature (a, e). Structure of the matrix built from multidimensional melting data (b, f). Columns of the data matrix show the UVRR spectrum at a single temperature (c, g). Rows of the data matrix show the melting curve at a single wavenumber (d, h).

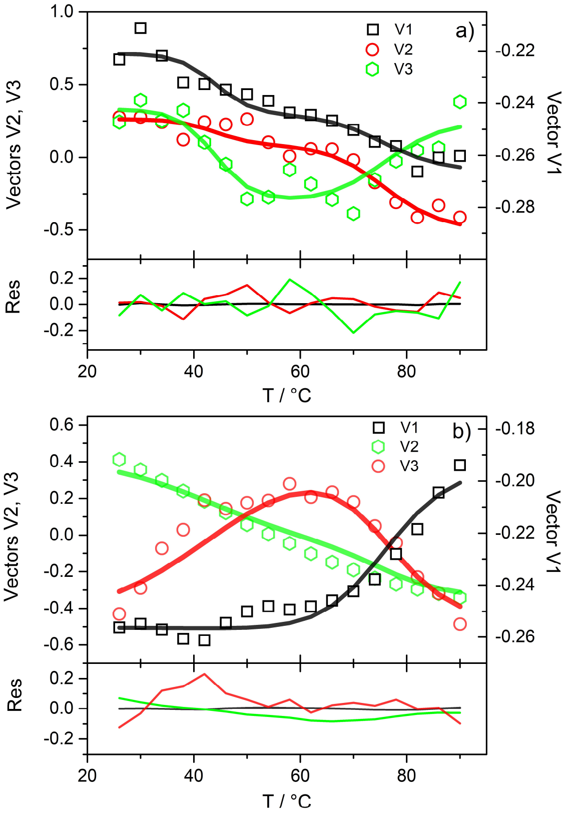

Figure S2. SVD analysis of UVRR data set for Tel22 K^+ aqueous solution, over the region I and II shown in Figure 1 of the manuscript. Panel (a) and (b): Vectors V1-V3 (full dots) as a function of temperature for Tel22. The lines represent the global non-linear fit of the data set to the expression of Equation (3) of the manuscript, concerning sequential

transitions between three spectral species: N ⇌ I ⇌ U. The plot of the corresponding residuals of the fit for the three vectors are reported at the bottom of each panel.

SVD analysis			
**Index**	**Vectors Autocorrelation Coefficient (V)**	**Singolar Value**	**Relative Variance**
**ZONE I 1390-1800 (cm^{-1})**			
1	0.83	3393	0.88
2	0.8	112	0.29
3	0.69	45	0.012
4	0.17	40	0.01
**ZONE II 2700-3900 (cm^{-1})**			
1	0.75	146782	0.9345
2	0.83	8860	0.056
3	0.69	358	0.0023
4	0.38	130	0.0008

Table S1. Parameters from SVD analysis of melting UVRR data for Tel22 aqueous solution used to decide the number of significant spectra: The autocorrelation of the vectors temperature dependent (V), the magnitude and the relative variance of each singular value. In general, for the autocorrelation, a cut-off of 0.8 is used [S1, S2], in our case we decided to reject the vectors with autocorrelation lower than 0.65. We used this criterion since, for experimental reasons, the step between each temperature was bigger than usual steps and thus all the vectors were noisier.

	**SVD shared fit**	
	Spectral range 1390-1800 & 2700-3900 (cm^{-1})	
	T_{m1} (°C)	44.1±2.5
	T_{m2} (°C)	76.0±3.0
**ZONE I** 1390-1800 (cm^{-1})	ΔH_1 ($kcal\cdot mol^{-1}$)	-56.1±3.5
	ΔH_2 ($kcal\cdot mol^{-1}$)	-44.6±3.4
**ZONE II** 2700-3900 (cm^{-1})	ΔH_1 ($kcal\cdot mol^{-1}$)	-18.8±3.5
	ΔH_2 ($kcal\cdot mol^{-1}$)	-38.5±3.0

Table S2. Thermodynamics parameters for the thermal melting of Tel22 aqueous solution obtained from a global fit on SVD vectors derived for both region I and II of Figure 1 of the manuscript. The fit performed by sharing T_{m1} and T_{m2} well represents the spectral species over both ranges.

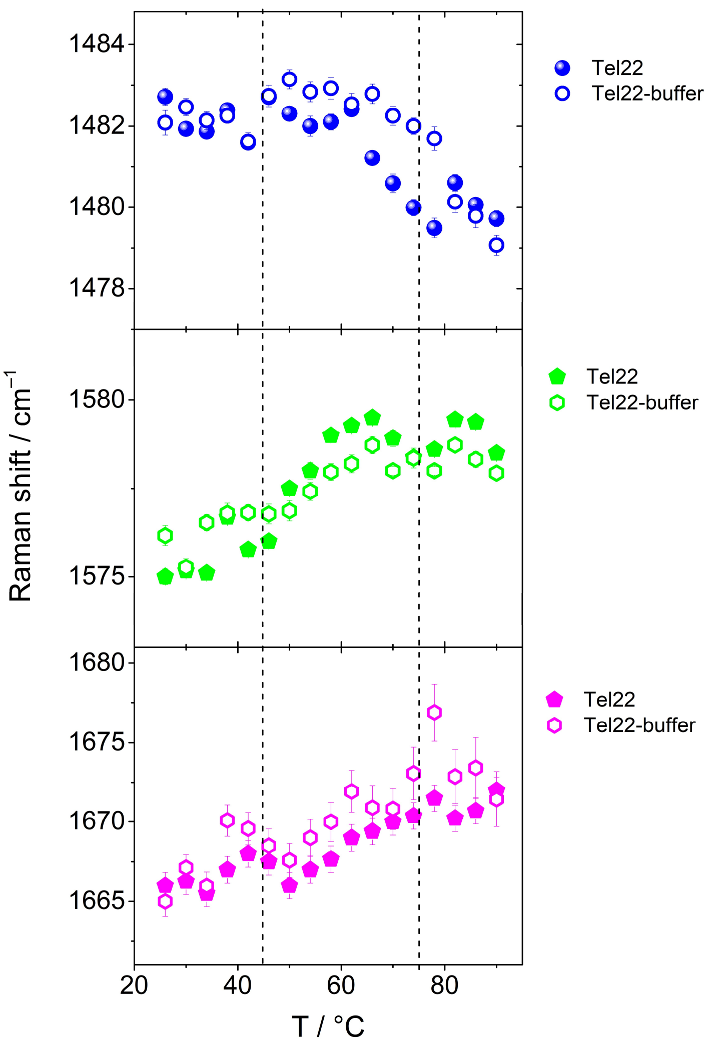

Figure S3. Raman shift of the vibrational group bands centered at about ν_{1482}, ν_{1575} and ν_{1666} cm^{-1} as a function of temperature. Fitting results corresponding to Tel22 and Tel22 solvent-free solutions are compared to test the robustness of the analysis.

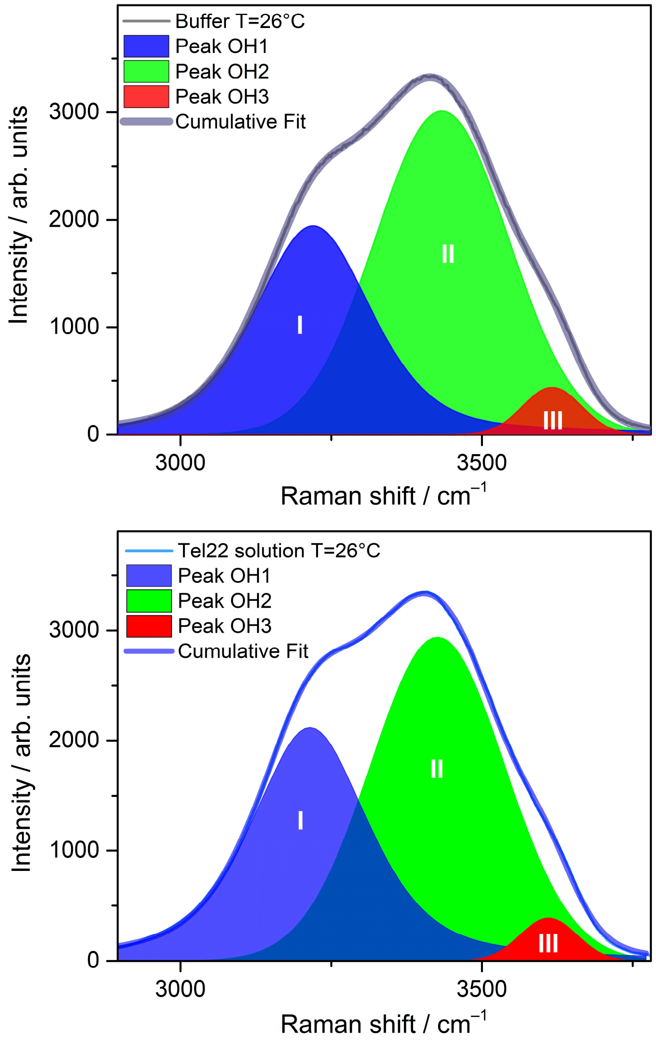

Figure S4. The total fit-curve (solid lines) and the single components (shaded areas) assigned to OH stretching modes represented for both buffer (top panel) and Tel22 solution (bottom panel) at the same temperature. Peak II and peak III are reproduced by gaussian functions, while peak I by a mixed lorentzian/gaussian function (coupling parameter ~ 0.6) [S3].

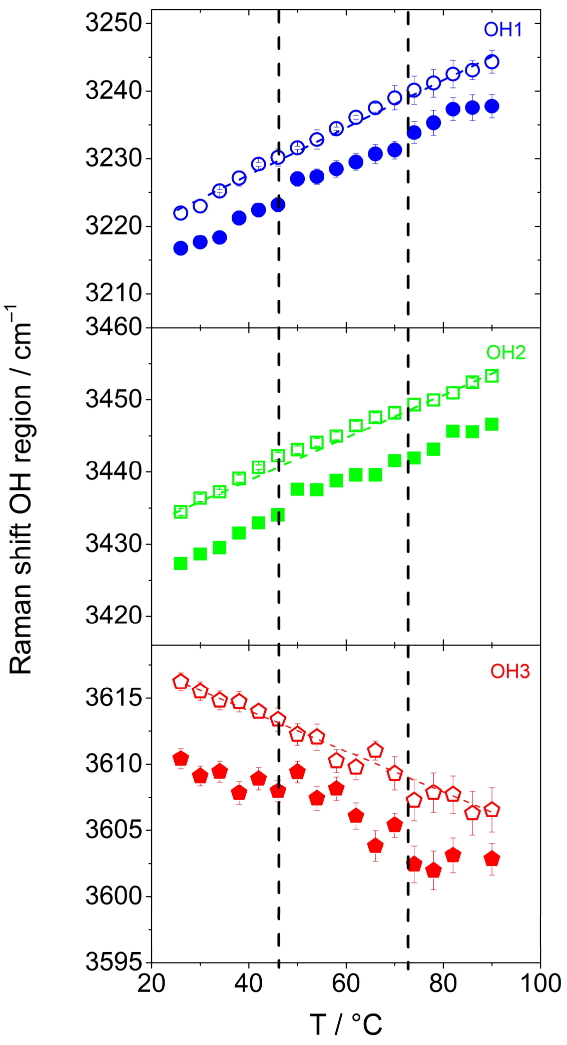

Figure S5. Raman shift of the OH stretching group bands of Figure S4, represented as a function of temperature: open and solid symbols refer to buffer and Tel22 solutions, respectively.

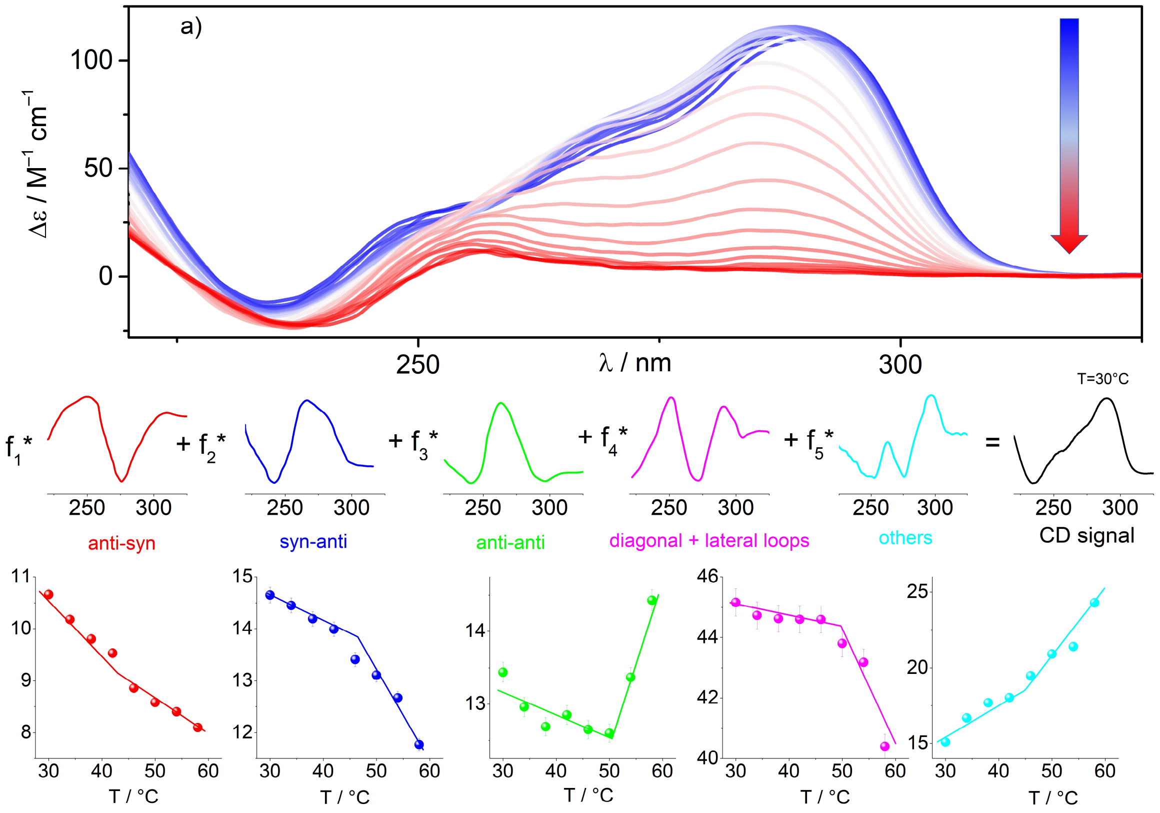

Figure S6. Temperature evolution (from 30°C to 82°C) of the CD spectra of a 45 μM Tel22 K^+ aqueous solution. Experimental profiles were analyzed by using the algorithm presented in Ref. [S4]. The percentage of secondary structure principal components, i.e. anti-syn, syn-anti, anti-anti, diagonal+lateral loops and other, are reported at the bottom as a function of the temperature, showing a kink between 44°C and 50°C, lines are only guides to the eye.

References

[S1] Gray, R. D.; Chaires, J. B. Analysis of multidimensional G-quadruplex melting curves. *Curr Protoc Nucleic Acid Chem.* **2011**, 45, 17.4.1-17.4.16.

[S2] Gray, R. D.; Buscaglia, R. and Chaires, J. B. Populated Intermediates in the Thermal Unfolding of the Human Telomeric Quadruplex. *J. Am. Chem.Soc*. **2012**, 134, 16834–16844.

[S3] Bottari, C.; Comez, L.; Paolantoni, M.; Corezzi, S.; D'Amico, F.; Gessini, A.; Masciovecchio, C.; Rossi, B. Hydration Properties and Water Structure in Aqueous Solutions of Native and Modified Cyclodextrins by UV Raman and Brillouin Scattering. *J. Raman Spectroscopy* **2018**, 49 (6), 1076–1085.

[S4] Del Villar-Guerra, R.; Gray, R.D.; Chaires, J.B. Characterization of Quadruplex DNA Structure by Circular Dichroism. *Curr. Protoc. Nucleic Acid Chem*. **2017**, 68, 17.8.1–17.8.16.
